# Single-balloon enteroscopy-assisted endoscopic ultrasound-guided gastroenterostomy for duodenal ascending part obstruction in stage IV pancreatic tail carcinoma

**DOI:** 10.1055/a-2641-9546

**Published:** 2025-07-25

**Authors:** Xue Sun, Xiaoming Wang

**Affiliations:** 1 159410 Department of Gastroenterology, Panzhihua Central Hospital, Panzhihua, China

A 50-year-old female with stage IV (T4NxM1) pancreatic tail carcinoma presented with gastric outlet obstruction (GOO) symptoms. Abdominal computed tomography demonstrated a stenosis at the junction between the duodenal horizontal part and proximal jejunum. Gastroscopic/colonoscopic localization attempts failed, but single-balloon enteroscopy (SBE; SIF-Q260, Olympus Medical Systems) identified luminal narrowing in the duodenal ascending part near the ligament of Treitz.

Given the patient’s cachectic state precluding surgical intervention, our department attempted SBE-assisted endoscopic ultrasound-guided gastroenterostomy (EUS-GE) ( Video 1 ). SBE reached the stenosis orally. Under fluoroscopic guidance, a 7-Fr nasobiliary tube was placed in the distal jejunum over a disposable guidewire for 500 mL methylene blue-stained saline infusion to distend the target segment. EUS identified a dilated jejunal lumen with gastroenteric wall distance <1 cm. A 19-G EUS needle punctured the jejunum to confirm luminal access. A 20 mm × 10 mm lumen-apposing metal stent (LAMS; Axios, Boston Scientific) was deployed at 100-W pure-cut mode.

Single-balloon enteroscopy-assisted endoscopic ultrasound-guided gastroenterostomy for duodenal ascending part obstruction.Video 1

After the procedure, the patient achieved marked palliation of GOO symptoms with a 5-kg weight regain observed within 40 days. Serial barium studies (postoperative day 4 and 2 months) confirmed sustained stent patency. Per treatment protocol, repeat EUS-GE was planned for potential afferent loop syndrome (ALS) secondary to complete stricture of the duodenal ascending part. This would involve creating a gastro-duodenal horizontal part anastomosis to decompress the proximal intestinal lumen. However, ALS did not occur during the follow-up of the patient.

To date, EUS-GE has been predominantly applied to gastric distal and duodenal proximal stenosis 1 2 3 4 . This represents the first documented case of SBE-assisted EUS-GE for duodenal ascending part obstruction secondary to pancreatic tail carcinoma. This case demonstrates the feasibility of SBE-assisted EUS-GE for duodenal ascending part-to-jejunal obstructions, but it is necessary of gastroenteric wall proximity (<1 cm) for safe anastomosis.

Endoscopy_UCTN_Code_TTT_1AS_2AB

## Notes

Endoscopy E-Videos https://eref.thieme.de/e-videos
*E-Videos* is an open access online section of the journal *Endoscopy* , reporting on interesting cases and new techniques in gastroenterological endoscopy. All papers include a high-quality video and are published with a Creative Commons CC-BY license. Endoscopy E-Videos qualify for HINARI discounts and waivers and eligibility is automatically checked during the submission process. We grant 100% waivers to articles whose corresponding authors are based in Group A countries and 50% waivers to those who are based in Group B countries as classified by Research4Life (see: https://www.research4life.org/access/eligibility/ ). This section has its own submission website at https://mc.manuscriptcentral.com/e-videos .
